# The traps of the internet in the covid era

**DOI:** 10.1192/j.eurpsy.2021.1253

**Published:** 2021-08-13

**Authors:** I.D. Rădulescu, L. Luca, V. Bokolas, V.D. Mărineanu, S. Rawat, A.B. Ciubară, A. Piotrowski, E. Vashdi, A. Ciubară

**Affiliations:** 1 Psychiatrist, “Elisabeta Doamna” Psychiatric Hospital, Galati, Romania; 2 Phd Student, Faculty of Medicine and Pharmacy, University “Dunarea de Jos”, Iasi, Romania; 3 Phd, Educational Scientist, Xenios Polis, Athens, Greece; 4 Phd, Psychologist, București University, București, Romania; 5 Phd, Psychologist, Military MIND Academy, Pune, India; 6 Md, Ph.d, Associate Professor, Faculty of Medicine and Pharmacy, University “Dunarea de Jos”, Galati, Romania; 7 Phd, Psychologist, University of Gdansk, Gdansk, Poland; 8 Phd In Physical Therapy, Yaelcenter, Haifa, Israel; 9 Md, Ph.d., Hab. Professor, Faculty of Medicine and Pharmacy, University “Dunarea de Jos” Head of Psychiatry Department, Senior Psychiatrist at ”Elisabeta Doamna” Hospital, Galati, Romania

**Keywords:** psycho-education, traps, Covid, Internet

## Abstract

**Introduction:**

The paper presents the results of one international study due by our team in the period of restrictions imposed by Covid-19, between March and June 2020.

**Objectives:**

To inform the population about the risks concerning the excessiv use of internet To prevent the effects of those behaviors which affects the global functioning of individuals

**Methods:**

Questionnaire applied to a number of 549 subjects, 18 to 60 years old, 217 from Romania and 332 from other European and Asian countries

**Results:**

The results allowed us to verify the assumption that there is a change in communications needs of individuals, as well gender and age differences in terms of time spent on the internet during the covid period.

**Conclusions:**

The issue of psycho-education regarding the awareness of dangers and the traps of the virtual era remain relevant.

